# V-Alert: Description and Validation of a Vulnerable Road User Alert System in the Framework of a Smart City

**DOI:** 10.3390/s150818480

**Published:** 2015-07-29

**Authors:** Unai Hernandez-Jayo, Idoia De-la-Iglesia, Jagoba Perez

**Affiliations:** Deusto Institute of Technology (DeustoTech), University of Deusto, Bilbao 48007, Spain; E-Mails: idoia.delaiglesia@deusto.es (I.D.-I.); jagobaperez92@opendeusto.es (J.P.)

**Keywords:** vehicular networks, smart cities, vulnerable road users

## Abstract

V-Alert is a cooperative application to be deployed in the frame of Smart Cities with the aim of reducing the probability of accidents involving Vulnerable Road Users (VRU) and vehicles. The architecture of V-Alert combines short- and long-range communication technologies in order to provide more time to the drivers and VRU to take the appropriate maneuver and avoid a possible collision. The information generated by mobile sensors (vehicles and cyclists) is sent over this heterogeneous communication architecture and processed in a central server, the Drivers Cloud, which is in charge of generating the messages that are shown on the drivers’ and cyclists’ Human Machine Interface (HMI). First of all, V-Alert has been tested in a simulated scenario to check the communications architecture in a complex scenario and, once it was validated, all the elements of V-Alert have been moved to a real scenario to check the application reliability. All the results are shown along the length of this paper.

## 1. Introduction

According to the European Commission Directorate-Generals for Mobility and Transport [1], in 2014, slightly more than 25,700 road fatalities were reported in the European Union. Although the number of road fatalities is substantially decreasing, the Transport White Paper [2] announced a strategic target for EU road safety for the period of 2011–2020: to reduce the number of road deaths by half. If the statistics are analyzed, in the period of 2010–2012, the number of cyclists killed increased by 6% from 2010 to 2012, being the only one road user group whose trend was worse. This is explained, at least partly, by an increase of the total number of cyclists on the roads. It can be said that cycling is a transport mode where relatively Vulnerable Road Users (VRU) interact with traffic of high speed and mass. When there is an accident, they suffer the most severe consequences in collisions with other road users because they cannot protect themselves against the speed and mass of the other party.

Based on these studies, crashes involving VRUs occur frequently in facilities designed for pedestrians and cyclists, such as pedestrian crossings, cycle tracks, and cycle lanes, that are close to or part of the common traffic infrastructures, that is, on the road. Therefore, the question is: How can we reduce the number of VRU crashes and the severity of their injures? Different solutions can be taken: improve the lane use planning and the road design, increase the visibility of the VRUs with to better lighting systems, stimulate the use of protective devices such as helmets and promote more training and educative courses among the VRUs.

However, apart from answers oriented to the construction or redesign of new infrastructures or passive solutions such as the use of helmets, an alternative approach that is gaining importance in developing countries is the development of solutions for mobility in the framework of Smart Cities. Although a Smart City can be a quite fuzzy concept, it can be said that a city can be considered *smart* if it has been provided with Information and Communications Technologies (ICTs) to enhance quality of life by improving safety and well-being in areas like resource consumption, cost reduction, and government and mobility, allowing the more effective and active participation of citizens.

In the framework of Smart Cities, designed mobility solutions are aimed to make better, safer, and more sustainable use of the road infrastructure through a better understanding of the state of the traffic, the location of vehicles and users, and the registration of events that occur during the mobility. These solutions combine the capacity and benefits of sensors, devices, physical infrastructure, and communication architectures with digital information systems and the ability to process large volumes of data through new software tools that can be executed on the cloud.

In this context, Intelligent Transportation Systems (ITS) emerge as a technological response to better monitoring and characterization of traffic. These systems allow at the same time to improve the use and efficiency of road infrastructure, as well as the safety of users, particularly those referred to as Vulnerable Road Users, such as pedestrians, cyclists, or bikers. Current ITS require the use of traffic cameras, variable signal panels, or inductive sensors to gather information about the traffic, meaning that they need to deploy physical devices on the infrastructure to obtain, send, and process data in a traffic management center. In opposition to these kinds of solutions focused on the use of infrastructure sensors, nowadays the rise of systems known as Floating Car Data (FCD), based on the collection of information from vehicles’ Global Positioning Systems via mobile terminals and the collaborative use of information through web platforms like Waze, allow drivers to provide and obtain useful information about traffic events without any sensor deployment on the road. These types of solutions based on FCD favor that users monitor the state of the infrastructure in a ubiquitous way, but their reliability depends on the number of vehicles and users that participate in reporting data and events.

In the framework of ITS, Cooperative-ITS (C-ITS) are systems that allow direct links between vehicles (V2V communications) or between vehicles and the infrastructure (V2I or I2V communications) in order to exchange information to improve active road safety and traffic management. These links are possible thanks to On-Board Units (OBU), C-ITS dedicated devices that enable these communication interfaces, and thanks to devices located on the infrastructure known as Road Side Units (RSU).

The importance of C-ITS technologies for public administration and the European Commission is reflected in the 2010/40/EU directive, where the EU recognizes the capacity of the C-ITS to improve actual traffic management systems and to drive the processes of implementation and deployment of these systems in European road infrastructures. After dozens of research and development projects such as Cooperative Vehicle-Infrastructure Systems (CVIS), Cooperative Systems for road safety- “Smart vehicles on smart roads” (SAFESPOT), Tomorrow’s Elastic Adaptive Mobility (TEAM), the massive deployment of the C-ITS system is closer. An example is the Memorandum of Understanding (MOU) signed by automotive industry and infrastructure organizations with the aim of starting to deploy solutions based on C-ITS by 2015 [3]. Public administrations are also working on the same direction, highlighting the agreement reached among Germany, Austria, and the Netherlands to deploy a corridor among these three countries equipped with C-ITS technologies [4]. It is also worth noting the announcement in February of 2014 by the National Highway Traffic Safety Administration (NHTSA), belonging to the United States Department of Transportation (USDOT), of its intention to take the necessary steps for the deployment of V2V cooperative systems in the coming years, particularly from 2017, for commercial vehicles.

In a C-ITS scenario, four types of communicating agents are considered: two mobile entities (OBUs and pedestrians), and two stationary entities (the RSUs and the central system, which could be referenced as a Traffic Management Center). These entities are able to run four classes of applications: active road safety, cooperative traffic efficiency, cooperative local services, and global Internet services. In each class, different applications and use cases are defined, where each agent can be considered a sensor that produces information. Depending on the application and timing restrictions, data exchange among referenced entities can be categorized as:

- Warning messages: these are defined as decentralized environmental notification messages and they can be sent out to each vehicle or RSU.
- Heartbeat messages or beacons: these messages are used by OBUs to report their position, speed, and identification to the RSUs in the form of FCD. Moreover, these messages are also used to keep updated information about traffic situations. For this, Wireless Access in Vehicular Environment (WAVE) defines Cooperative Awareness Messages (CAMs), which are broadcasted periodically by each vehicle.
- Non-safety messages: these are used to enhance driver information and comfort (tourism information, Internet access, navigation aid, and so on).

In the field of cooperative ITS services, a huge variety of applications and use cases that are focused on increasing user safety can be described. Taking into account strategic, economical, and organizational requirements, and system capabilities and performances as well as legal and standardization requirements, the European Telecommunications Standards Institute Technical Committee on ITS has defined a basic set of applications to be used as a reference for developers [5]. Among them, Vulnerable Road User Warning aims to provide warnings to vehicles of the presence of vulnerable road users, e.g., pedestrians or cyclists, in case of dangerous situations and it also alerts the VRUs about the presence of nearby vehicles.

Therefore, following the requirements provided by the ETSI, in this paper we present V-Alert, the C-ITS system defined in the frame of Smart Cities that allows the use of vehicles as mobile sensors that share their positions, speed, and direction in form of FCD with the VRUs (V-Alert is devoted to cyclists in this case), warning each other about their locations in order to avoid collisions. The architecture described in this approach is based on a centralized system that deploys specific wireless vehicular communications, mobile communications, and a cloud computing software architecture that manages all the information obtained from the users (drivers and cyclists). The system has been deployed and tested in a real location and it has also been simulated in different scenarios in order to check how the performance of the communications and the application could be in the worst conditions. Currently, in the scope of Intelligent Cooperative Sensing for Improved traffic efficiency (ICSI) project, similar applications are under development using a decentralized approach in order to improve the performance of safety and security applications.

The paper starts with a brief description of related applications that focus on the detection of VRU. Then, in Section 3, the simulated scenario of V-Alert is described and tested. After the validation of the simulation, the V-Alert application and architecture is described and finally tested in Section 5. The paper ends with the conclusions and future steps to improve the application.

## 2. Related Work

Applications to detect VRU are not new in literature. For many years, systems that have used different techniques to detect mainly pedestrians have been deployed. Image recognition is likely the scientific field in which more efforts have been made to minimize the impact of possible accidents between pedestrians and vehicles. At [6], Takahashi *et al.* implemented a urban road user classification framework using local feature descriptors and hidden Markov models (HMM) to detect pedestrians, bicyclists, motorcyclists. In the approach defined by Fardi *et al.* [7], a multi-sensor system based on sensors near infrared cameras and Wireless Personal Area Network (WPAN) communications devices is defined, providing only a VRU tracking range of 60 m and no matrix about the communication performance.

Other applications are based on radar systems. For example, Heuel *et al.* [8] were able to measure target range and radial velocity thanks to a 24 GHz radar deployed on board the vehicle. On the other hand, Schaffer *et al.* [9] propose a more complex system that uses a secondary radar schema to detect and localize vulnerable road users, infrastructure, and other vehicles equipped with transponders in order to improve traffic safety, even under Non-Line of Sight (NLOS) conditions.

Applications like [10,11,12] have been configured based on data interchange between vehicles and VRU using nomad devices, but using, in all the cases, short-range communications platforms and not combining short- and long-range links like in V-Alert, providing more coverage area and then, more reaction time for drivers and VRUs. In other cases, these approaches are not considered cooperative applications due to not supporting multiple road users with communication availability.

Such sensors based applications nevertheless do not operate correctly at night or in poor visibility conditions, in bad weather conditions, or if the VRU is not close enough or in NLOS position in respect to the sensor. Moreover, many of these systems are focused only on the detection of the VRU from the perspective of the vehicle and not from the VRU, meaning that only the vehicle is advised about the presence of the VRU and then only the driver can perform a preventive maneuver. Moreover, these systems require the on-board deployment of not only complex sensor systems but also of processing equipment able to analyze huge amounts of data in real time. V-Alert has been designed using the characteristics and data processing properties of common devices, such as smartphones and non-sophisticated servers, in order to minimize the requirement of complex and expensive devices. This approach also has been selected to achieve a fast market penetration of these kinds of applications without the implication of vehicle manufacturers, which is needed if any hardware must be deployed on board. It is true that in V-Alert Institute of Electrical and Electronics Engineers (IEEE) 802.11p communication units have been used, but it is expected that this technology will be the *de facto* one to provide connectivity to the vehicles combined with long-range communications links like Long Term Evolution (LTE) [13].

## 3. First Approach: Simulated Scenario

### 3.1. Simulated Architecture

As it is mentioned in the introduction, the main target of V-Alert is to avoid accidents that involve Vulnerable Road Users in an urban scenario. Although the proposed solution in this work is completely functional, it has only been possible to test on a small scenario. Trying to obtain the results of the behavior of V-Alert in more complex scenarios, we decided to test this application in a simulated scenario with more vehicles (four), more cyclists (eight), and by modeling the communications parameters, which are critical to the application’s reliability.

**Figure 1:**
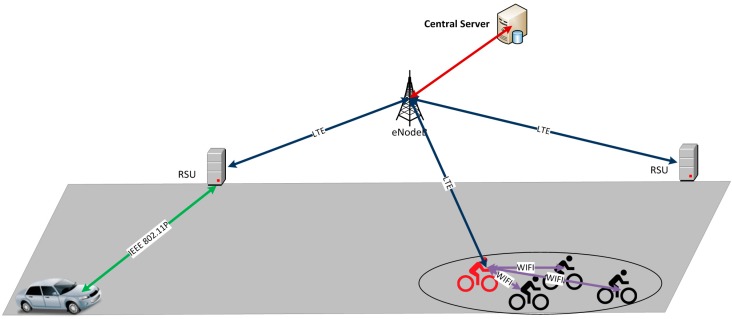
Description of the simulated architecture.

Figure 1 outlines the architecture designed for validating the performance of V-Alert in a FCD system by means of simulation. This architecture allows the exchange of information between mobile sensors (referenced in the paper as vehicles and bikes) thanks to a central server that has a global overview of the road scenario.

This multi-technology architecture allows the execution of the three main phases of V-Alert, which are: (1) data collection; (2) centralized information management; (3) awareness messages dissemination.

(1) Data collection: this phase comprises everything related to sending information to the central server. Therefore, on one hand, as every vehicle acts as a sensor sending periodic Cooperative Awareness Messages (CAMs), each RSU is able to know the position information of all the vehicles that are in its coverage area and sends updated information periodically to the central server. On the other hand, bikes are also sending periodic messages with their position information inside the Wi-Fi network; consequently, the Master Bike is able to aggregate the information about its group and sends it to the central server through LTE technology.
(2) Centralized information management: the reception of updated information of the road situation by the central server allows checking which vehicles are in the route of a bike group and deciding to inform the vehicles about the presence of the bikes and to inform the bikes about the position of the vehicles.
(3) Awareness message dissemination: this phase is in charge of sending the information of the vehicles to the bike group and the information of the bike group to the vehicles. Thus, the central server sends the information of the bike group to the RSUs, where the interested vehicles are in their coverage area through LTE, and the RSUs disseminate this information though IEEE 802.11p with the aim of being received by the interested vehicles. At the same time, the central server sends the information of the vehicles that are approaching to the Master Bike by LTE and the Master Bike disseminates this information to the rest of the bikes through Wi-Fi.

### 3.2. Simulation Setup

The main objective of the simulation is to show the feasibility and performance of the previously proposed architecture for V-Alert. Therefore, this architecture is evaluated using the discrete-event network simulator NS-3 [14], which is an open-source and validated simulator widely used by the research community. Furthermore, NS-3.21 provides models for assessing heterogeneous vehicular networks, including models for short-range communication networks such as Wi-Fi and IEEE 802.11p and for cellular networks such as LTE. For generating the mobility traces to be driven by vehicles and bikes during the NS-3 simulation, the Simulation of Urban Mobility (SUMO) traffic simulator [15] is used. The simulated scenario is the suburban area in Bilbao where there are usually multiple groups of bikes and the flow of vehicles is not continuous. Vehicles and bikes move during 600 s on a road of 5 km. Table 1 details the simulation parameters used in the evaluation.

**Table 1:** Simulation parameters.

Type	Parameter	Value
Vehicles	CAM frequency	1 Hz
RSU	TMC update frequency	CAM message received by vehicle
Bike	Master Bike update frequency	1 Hz
Bike Master	TMC update frequency	1 Hz
Scenario	Type	Suburban
	Number of vehicles	4
	Number of bikes	8
	Vehicle speed	10–70 km/h
	Bike speed	25–30 km/h
IEEE 802.11p Network	Bit Rate	3 Mbps
	Bandwidth	10 MHz
	Frequency band	5.9 GHz
	Maximum tx power	21 dBm
	Propagation model	Nakagami
IEEE 802.11b Network	Bit Rate	1 Mbps
	Bandwidth	20 MHz
	Frequency band	2.4 GHz
	Maximum tx power	16 dBm
	Propagation model	Nakagami

### 3.3. Metrics and Results

The following metrics are considered in this study:

- Bike reception delay (ms): the time elapsed between the message transmission by a vehicle and the message reception for a given bike.
- Vehicle reception delay (ms): the time elapsed between the message transmission by the Master bike and the message reception for a given vehicle.

**Figure 2:**
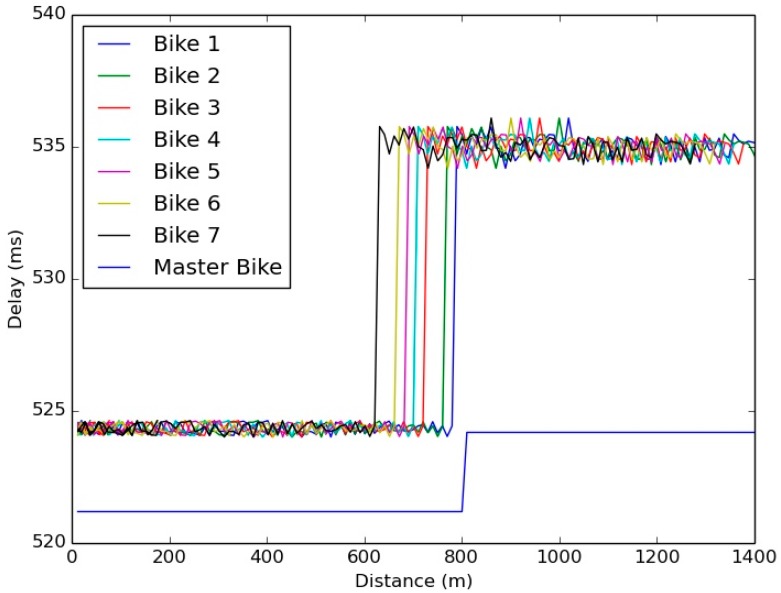
Bike reception delay.

**Figure 3:**
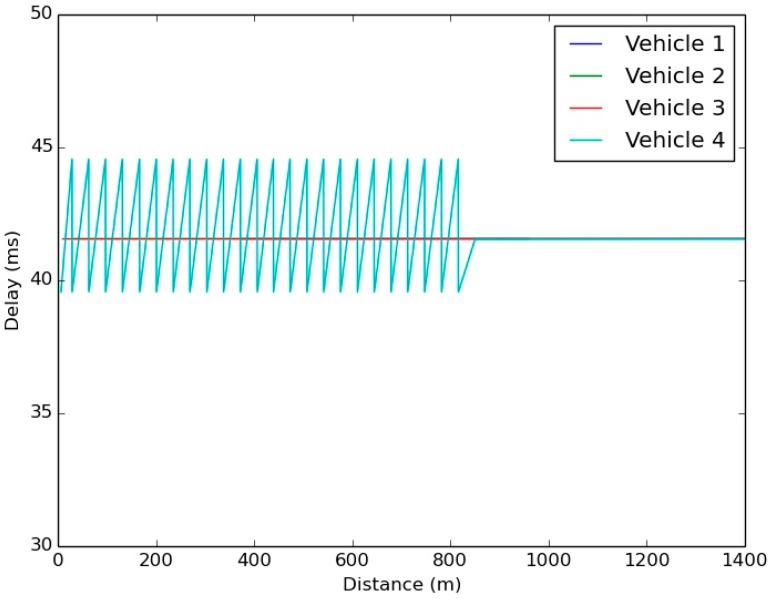
Vehicle reception delay.

The validation of the simulation’s results is necessary to deploy the application in real environments. The reliability depends on the delay of a message to reach its destination. Therefore, Figure 2 shows the average delay of a message to go from a vehicle to a bike depending on the distance between the vehicle and that bike. As we show in the Figure 2, the time a message needs to reach the Master Bike is less than the rest of the bikes because the Master Bike is the one in charge of disseminating the vehicle’s message to the rest of bikes through the Wi-Fi network. Figure 3 shows the Vehicle reception delay, which is almost constant for all distances.

Comparing the delay results of Figure 2 and Figure 3, it is observed that the bike reception delay is in the range of 522 ms and 535 ms, but the average vehicle reception delay is 40 ms. This difference is owed to the route each message has to follow and the Traffic Management Center (TMC) updating frequency of the RSU and the Master Bike.

The bike reception delay is defined by Equation (1) for the Master Bike and Equation (2) for the rest of bikes in the group

(1) $$D_{Bike\;Master}=\;D_{V-RSU}+\;D_{RSU-TMC}+\;D_{TMC-BM}$$

(2) $$D_{Bike}=\;D_{V-RSU}+\;D_{RSU-TMC}+\;D_{TMC-BM}+\;D_{BM-B}$$

where

- *D_V-RSU_* is the time from the message being generated in the vehicle until the RSU receives the message.
- *D_RSU-TMC_* is the time that passes from when the RSU receives the message from the vehicle until it is received by the TMC.
- *D_TMC-BM_* is the time that passes from when the TMC receives the message from the RSU until it is received by the Master Bike.
- *D_BM-B_* is the time that passes from when the Master Bike receives the message from the TMC until it is received by the bikes that are in the Wi-Fi network.

The vehicle reception delay is defined by Equation (3)

(3) $$D_{Vehicle}=\;D_{BM-TMC}+\;D_{TMC-RSU}+\;D_{RSU-V}$$

where

- *D_BM-TMC_* is the time that passes from when the message is generated in the Master Bike until it is received by the TMC.
- *D_TMC-RSU_* is the time that passes from when the TMC receives the message of the Master Bike until it is received by the RSU.
- *D_RSU-V_* is the time that passes from when the RSU receives the message from the TMC until it is received by the vehicle.

**Figure 4:**
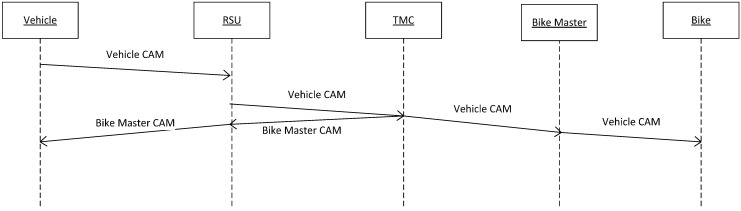
V-Alert sequence diagram.

The difference is generated because the TMC only sends awareness messages to the RSUs and to the Master Bike when it receives a message from a vehicle. Since the bike group is updating the information to the TMC through the Master Bike with 1 Hz frequency, if there is no vehicle in the RSU coverage area, there is no need to send the position of the bikes to the RSU. Consequently, as the RSU updates its information to the TMC with a frequency of 1 Hz, as it is shown in Figure 4, there is a delay since the RSU receives the message from the vehicle until it transmits the message to the TMC. However, this delay does not exit from the Master Bike to the TMC due to the Master Bike generating a new message with the aggregated information of the bike group.

## 4. Vulnerable Road User Application: Cyclist Warning System

### 4.1. System Overview

As it is described in previous sections, the goal of this work is to check the performance of the V-Alert application in a real scenario inside the city. To perform this test, only two vehicular communication gateways with IEEE 802.11p interface are available, so the scenario that can be implemented is smaller than the simulated one, but it can be really useful to obtain data about the application and the communication reliability between cyclists and vehicles that are equipped with different communication technologies.

**Figure 5:**
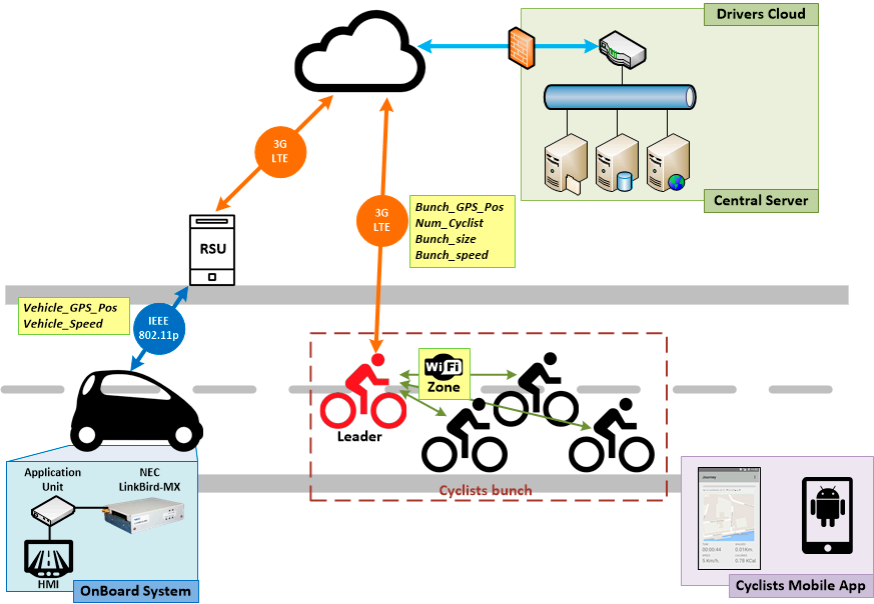
Description of the system architecture.

The deployed V-Alert platform is shown in Figure 5, where a hybrid communication network is implemented in order to intercommunicate all the agents involved in V-Alert: cyclists (that play the role of Vulnerable Road Users), vehicles, and the central server known as Drivers Cloud. The target of V-Alert is to warn cyclists and vehicles about each other’s the presence when they are close. Therefore, the Human Machine Interface (HMI) onboard the vehicle and the application that cyclists setup in their smartphones must display the relative position of the vehicles and cyclists, meaning if the vehicle is in front, left, right, or back in respect to the position of the cyclist and the distance to the point at which the cyclist and vehicle will intersect. Obviously, the main critical aspect of this application is that both cyclists and vehicles must receive this information with enough time to react and perform the appropriate maneuver to avoid risky situations.

It has been considered in a scenario in which several cyclists can ride alone or together in a bunch. In this situation, the application provided to the cyclists is ready to create a private Wi-Fi network, in which one cyclist plays the role of the leader and he is in charge of sending and receiving information from services deployed on the Drivers Cloud. The leader sends information about the bunch: GPS position (only of the leader), number of cyclists, size (calculated from GPS positions of further cyclists in the bunch), and the average speed of the bunch. At the same time, the leader broadcasts information received from the Drivers Cloud about the vehicles’ GPS positions close to the cyclists. The communication link between the cyclists and the Drivers Cloud uses a mobile data link that can be 3G or LTE, depending on availability.

On the vehicle’s side, the information about the speed and GPS position of each one is gathered by a Road Side Unit (RSU) through an IEEE 802.11p link. Then, the RSU is connected to the Drivers Cloud using a 3G or LTE link. This approach has been chosen in order to check the communication reliability when the V-Alert application reliability relies on a hybrid communication network and not only on a mobile one. The transfer data delay in these links is critical so this is the parameter that has been tested in the proposed scenarios.

### 4.2. System Architecture

**Figure 6:**
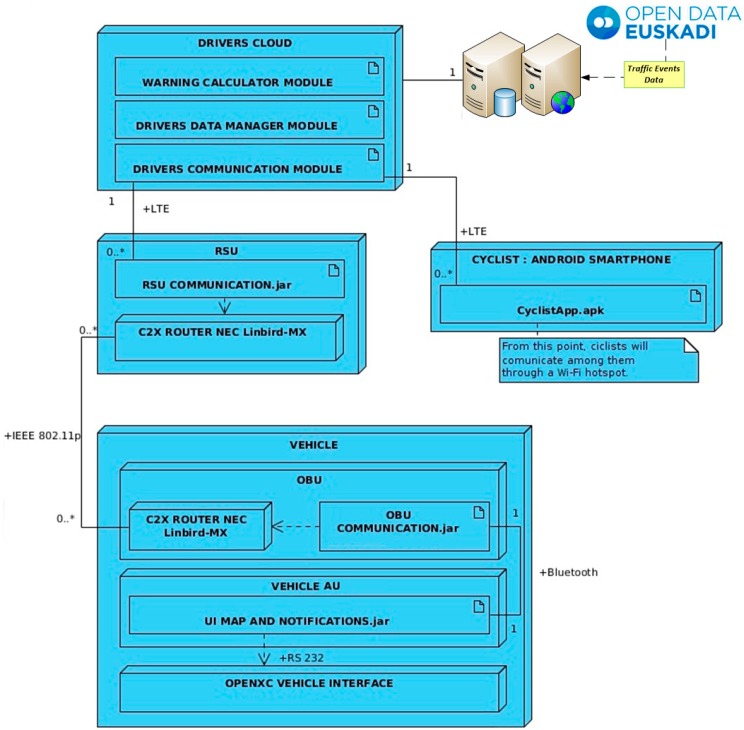
V-Alert software architecture.

To implement V-Alert architecture and services, the most flexible and open design has been chosen for the interconnection of the different subsystems. All the data generated by cyclists and vehicles are stored and transmitted in a plain format for being easily manipulated by all the agents involved in the system. This approach makes the development of different usecases and applications easy. The complete system is made of four different components: the Drivers Cloud, the RSU located on the infrastructure, the OBU deployed on a vehicle, and the smartphones of the cyclists. Figure 6 shows all the components of the V-Alert system, as well as its main software modules and the communication links that allow the data exchange among them.

The core of the system is deployed in the cloud server known as Drivers Cloud, where data provided from both cyclists and vehicles are processed and stored in a database. This server has been designed in a scalable way, where adding new modules is easy if they are required. The modules designed for the V-Alert application are the Warning Calculator Module, the Driver’s Data Manager’ and the Driver’s Communication Module, which are described at Section 4.5.

As seen in Figure 6, the architecture is also ready to access to open data platform, for example, as in Open Data euskadi. This is the database managed by the Basque Government in which information about traffic events can be found in real time. This website provides a description and geolocation of these events in a eXtensible Markup Language (XML) format that can also be useful for the drivers, but this implementation is out of the scope of the work described in this paper.

The connection between the RSU and the cyclists with the Drivers Cloud will be established with a LTE or 3G link, depending on availability, while vehicles are connected to the RSU through a specific vehicular wireless standard:

(a) In the case of cyclists, an Android application running in a smartphone has been developed. From this application, a register in the Drivers Cloud will be updated with the current position of a cyclist or a bunch of cyclists. All of them will receive data about the position of closing vehicles and/or other different events that can happen on the road (those that can be obtained from an open data base such as Open Data Euskadi). As it has been introduced before, V-Alert also has been designed to consider a bunch of cyclists that ride together on a route. In this case, a private Wi-Fi network is created among the cyclists where only one plays the role of leader and he is in charge of forwarding and receiving information to and from the Drivers Cloud.
(b) Vehicles access the Drivers Cloud through the RSUs settled in the infrastructure. V-Alert is a centralized C-ITS system, where all the information is saved and processed at the Drivers Cloud and the RSU only acts as a communication gateway, deploying an IEEE 802.11p interface to communicate with the vehicles and a LTE interface to connect with the Drivers Cloud. On the vehicles, the OBU implements the communication with the RSU and also shows the messages received by the RSU in the HMI of the vehicle. As it is described in the next section, the OBU is also ready to collect information from the electronic systems of the vehicle through an OpenXC interface and from the GPS of the vehicle.

### 4.3. Drivers Cloud Server

Drivers Cloud is based on a virtual machine executing a set of Java applications, with an embedded web server developed using *Jetty* technology, which are continuously listing HTTP/1.1 messages through a POST method. Drivers Cloud is in charge of receiving and processing data from vehicles and cyclists. Due to the huge amount of information that can be generated in this scenario, the server carries out a continuous update of the database in order to maintain in memory only those data and events that are relevant and that can be required for future analysis.

As it is shown in Figure 6, Drivers Cloud has been designed following a modular architecture in order to make its scalability easy in case more modules or service or required. In the scope of V-Alert, only three modules are needed:

- The Drivers Communication Module is in charge of listening to drivers’ position messages, filtering incorrect messages, and parsing and sending them to upper layers. It is composed of a Jetty-powered server which contains two controllers called handlers that process any message that is received by the server. Another handler is in charge of applying a defined message filtering policy in order to accept only POST messages and to reject those messages whose payload is wrong or corrupted. Messages are stored a maximum of two minutes in order to avoid memory overload.
- The Driver Data Manager is the module responsible for storing the messages that are accepted by the Drivers Communication Module, and making these data accessible to upper layers. When a message from a vehicle arrives to this layer, it is verified if a register of this vehicle already exists. If so, the register is updated with the newest information and if not, a new register is created. As it was mentioned before, in order to avoid the overload of redundant information, if a register of a vehicle is not updated in a certain amount of time and this vehicle has not produced a significant message, its register is deleted from the database. The registers of vehicles and cyclists that are stored in the server contain information about: an identifier of the device, its GPS position, heading, IP address, timestamp, and, in the case of being a bunch of cyclists, the number of cyclists in this position. This number is quite important in order to alert the vehicles about the size of the bunch and the area that they occupy on the road.
- The Warning Calculation Module is the core of the Drivers Cloud. This module compares data of both vehicles and cyclists for finding the distance between them. If one vehicle is closing in to a cyclist or bunch of cyclists, this module generates a high priority warning message that is broadcasted to the affected cyclists and vehicles.

### 4.4. Vehicle On-Board Unit and Road Side Unit Systems

**Figure 7:**
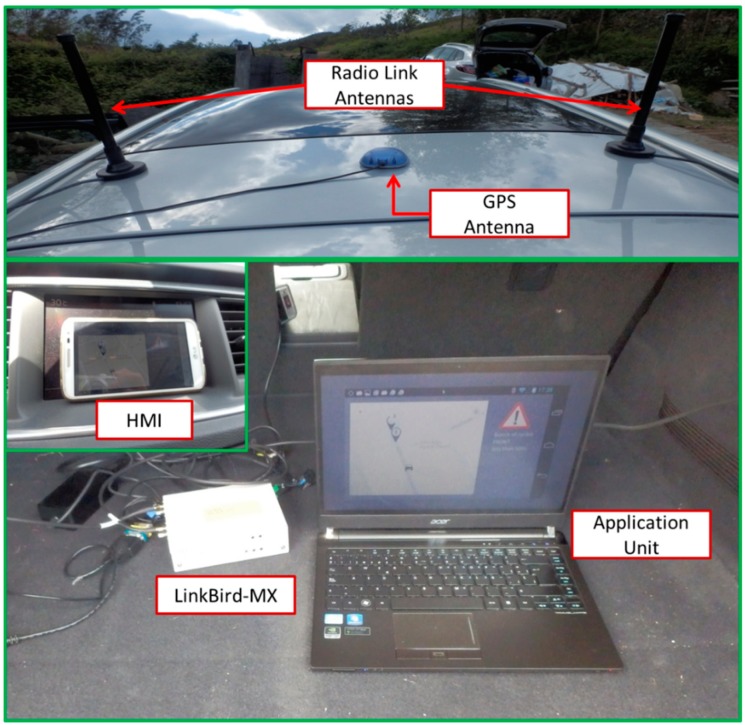
Hardware set-up.

As it is described at Section 4.1, the communication between the OBU and the RSU is performed thanks to an IEEE 802.11p radio link. In both entities, the same hardware has been deployed running as OBU or as RSU. The selected hardware is LinkBird-MX, which is an embedded Linux machine based on a 64 bit Microprocessor without Interlocked Pipeline Stages (MIPS) processor working at 266 MHz. Beside an IEEE 802.11p interface, these modules are equipped with an Ethernet connector that is used to communicate with the application unit (the one that runs the applications in a regular PC), a GPS interface, and other interfaces such as Controller Area Network (CAN) or RS-232. Figure 7 shows the hardware set-up used in the tests that have been carried out in the described scenarios.

Although LinkBird allows the selection of two-channel bandwidth, in these tests 10 MHz bandwidth has been selected instead of the 20 MHz one usually used by 802.11a devices, in order to minimize the multipath delay spread and the Doppler effect that appears in mobility and highway scenarios. Moreover, in order to maintain sufficient reliability of the data transfer in a one-hop scenario, the lowest bitrate has been used, which is 3 Mbps (bitrates from three to 27 Mbps are available at IEEE 802.11p standard), so also the lowest coding rate (1/2) with Binary Phase Shift Keying (BPSK) modulation has been used to transmit data packets. Along with the communication modules, two antennas whose characteristics fit well with vehicular applications are provided. One antenna is tuned to the 178 Control Channel (CCH) frequency (5.890 GHz) and the other one to the 180 Service Channel (SCH) frequency (5.9 GHz). Technical characteristics of the hardware setup are shown in Table 2. The RSU also deploys a mobile communication interface which links this system with the Drivers Cloud that is deployed in a remote server.

**Table 2:** Hardware set-up.

LinkBird-MX		Antenna	
Parameter	Values	Parameter	Values
Frequency	5725–5925 MHz	Model	ECO6-5500
Bandwidth	10 MHz	Frequency	5.0–6.0 GHz
Tx Power	21 dBm	Gain	6 dBi
Bitrate	3 Mbps	Radiation	Omni-directional

**Figure 8:**
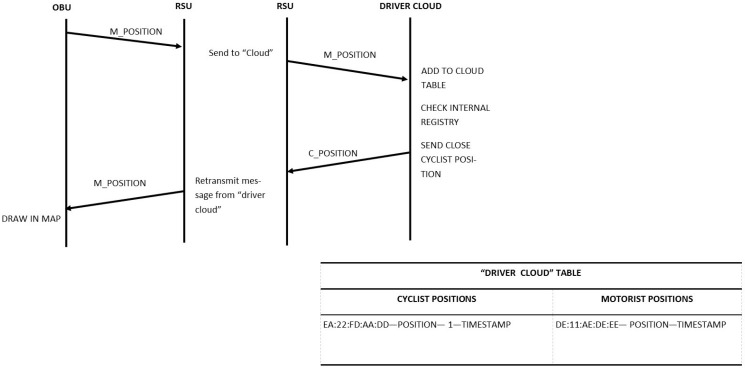
Communication protocol between OBU, RSU, and the Drivers Cloud.

To update the driver about the position of closing cyclists, the communication protocol shown in Figure 8 has been defined. The Warning Calculator Module deployed on the Drivers Cloud reports a warning message to the driver if there are some cyclists at a distance of less than 1 km. This geographical filter avoids overloading the driver with irrelevant information that can distract and endanger him or her.

As can be seen in Figure 8, there is a continuous data transfer from vehicles and the Drivers Cloud through the RSU. As an example, the format of the messages sent by the cyclist leader to the Drivers Cloud is shown in Table 3. In this message, the position of the cyclist bunch is reported to the server beside the number of the cyclists in the bunch (five cyclists are used in the example). In addition, this table reports the information about the vehicle with the identifier DE:11:AE:DE:EE, which is around 70 m from the bunch and is approaching at 15 m/s. With this data and as it is described in Section 4.5.1, vehicles and cyclists can also obtain information about the relative position of the other (front, left, right, or back).

**Table 3:** Message format interchanged between cyclists and the Drivers Cloud.

Message from the Bunch Leader to the Drivers Cloud							
hwAddr	Latitude	Longitude	Alt.	Heading	Speed	Components	Timestamp
EA:22:FD:AA:DD	43.2585269	−2.9377635	8	67	5	5	2 February 2015 10:39:40.433
Message from the Drivers Cloud to the Bunch Leader							
DE:11:AE:DE:EE	43.258146	−2.937094	6	280	15	–	2 February 2015 10:39:38.581

**Figure 9:**
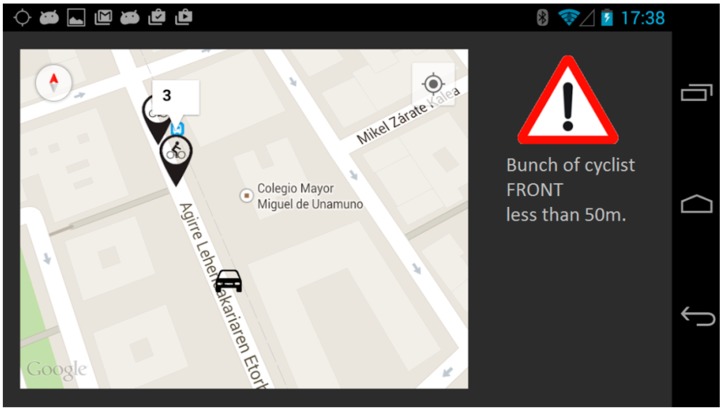
Human Machine Interface onboard the vehicle.

To display this information to the driver, the User Interface (UI) Map and Notifications application has been designed and deployed on the application unit (AU) that is connected to the LinkBird-MX on the OBU. It provides visual information to the driver about the cyclists’ position or other events that can occur on his route. The driver can access to this information thanks to a HMI that can be deployed on any Android device that is connected to the AU through a Bluetooth connection. In this prototype, the selected device is a tablet. This HMI consists of a graphical interface with a map (Figure 9) in which the cyclists and/or events are shown and there is an audible alarm that alerts the driver when a cyclist or a risky event is located closer than 100 m to him. Then, the driver has enough time to react and make the appropriate maneuver.

At the same time, in the OBU, several activities are performed in a parallel way in order to maintain the communication with other vehicles, cyclists, or RSUs:

1. Every 2 s, the geolocation of the vehicle is obtained thanks to a GPS module that is connected to the LinkBird-MX. Afterward, a message with this position is sent through a SHB (Single Hop Broadcast) message to the nearest RSU, which captures the message and addresses it to the Drivers Cloud.
2. The LinkBird-MX module sends and receives AddressMessage messages from other OBUs and RSUs, and stores their locations in an inner table. These GPS positions can be used by the OBU to report georeferenced information to these other agents. This means that the developed application is also ready to work in a Vehicle-to-Vehicle scenario in which the RSU would not be required.
3. On the AU, incoming messages are processed in the OBU communication application and shown to the driver through the HMI depending on their nature:
  - Cyclists’ position: an icon is drawn on the map with information about their relative position with respect to the vehicle (front, left, right, or back), the number of cyclists in the group, and the remaining distance until crossing with them.
  - Other notifications. The application shown in this paper is ready to report drivers’ information about events on the road. To feed the system, open data platforms with real-time information about the traffic can be used.

As it has been explained before, RSU works like a gateway between the vehicles and the Drivers Cloud, performing two main tasks:

1. The RSU receives messages from the OBU and addresses them to the Drivers Cloud: the RSU is constantly listening to messages, forwarding vehicles’ positions to the Drivers Cloud, and feeding the Warning Calculation Module to calculate the relative position of vehicles and cyclists.
2. The RSU receives messages from the Drivers Cloud and addresses them to the OBU: when a message is sent from the Drivers Cloud, first the header is read and depending on the type of message, (1) it will be addressed to the OBUs that are in the covered area of the RSU; or (2) as if the RSU were connected to a variable signal panel, the RSU is also ready to interact with these kinds of systems that are already deployed on the infrastructure and are part of an Intelligent Transport System.

### 4.5. Cyclist Mobile Application

**Figure 10:**
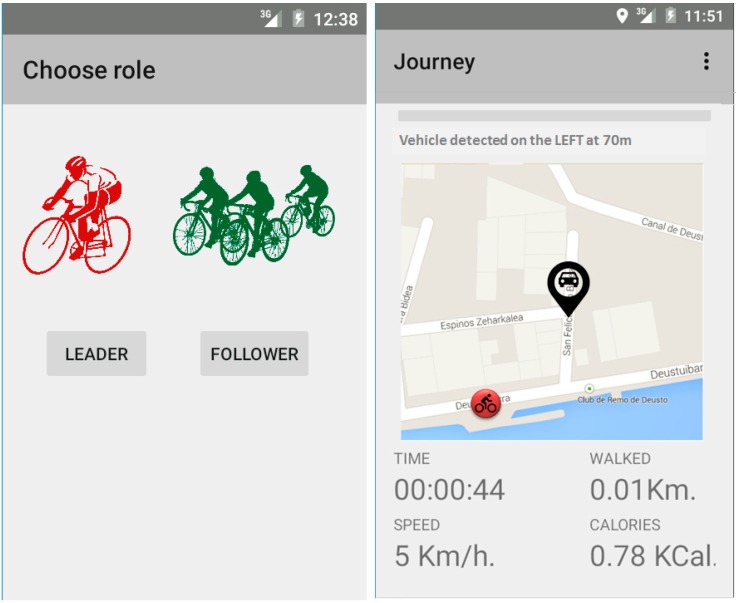
Cyclist application interface.

To be able to locate the cyclist, it is necessary to broadcast their position to the Drivers Cloud. The Android application for cyclists provides all these data (Figure 10), and it will also show a view of the road he is riding with the location of the nearby vehicles. Thus, the cyclist will be able to place himself/herself in a better position and be ready for being overtaken by a car. This interface has been designed taking the common sport applications that cyclists can use to monitor and record their activity as examples, displaying information about speed, distance, time, *etc*. Although the application is able to receive information about the surrounding vehicles up to 100 m, trying to not distracting the cyclist with unnecessary alarm messages, a warning signal is displayed only with a vehicle that is less than 20 m from the cyclist.

**Figure 11:**
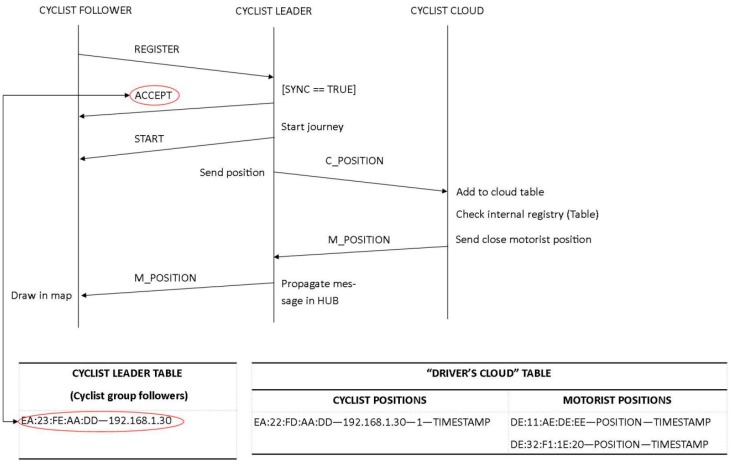
Communication protocol between cyclists and the Drivers Cloud.

As it is shown at Figure 10, before running the application, cyclists have to choose between two different modes of operation: individual or bunch ride. Both of them will show the same information to the cyclist, but they work in a different way:

- Individual ride: the user sends messages with his or her position through HTTP1.1 to the Drivers Cloud. Messages coming from the Drivers Cloud will be sent through a socket by the 8080 port by default to the cyclist’s device (Figure 11).
- Bunch ride: the leader of the bunch manages the messages coming from the Drivers Cloud. The other cyclists receive the positions and notifications from the leader thanks to the private Wi-Fi network that is setup at the beginning of the ride. First, the leader creates a virtual hub in which the other cyclists (that is, the followers) connect and communicate through a socket set up at port 1099 (Figure 11). When a device is trying to connect to the virtual hub, the follower sends a message asking the leader to get into the bunch. If the leader accepts the connection, a confirmation message is sent to the follower. Once all the cyclists’ devices are synchronized, the leader notifies the beginning of the ride. The leader’s device sends all data related to the bunch to the Drivers Cloud and it also receives and addresses the messages received from the cloud to the followers, who only listen to messages coming from the leader. In this first version of the application, if the leader leaves the group, the remaining cyclists have to reconfigure the application.

#### Relative Position Algorithm

As described previously, the main goal of V-Alert is to give advice to vehicles and cyclists about their relative positions in order to avoid accidents. However, in some situations, as, for example, in an intersection, distance is not enough information to avoid a collision because the cyclist also needs to know if the vehicle is close by his/her right or left or if it is ahead or behind. Obviously this information is useful for the driver, so this algorithm is running in both interfaces, the OBU and the cyclist’s mobile application, the description of which is included in Algorithm 1 where the relative position of vehicle B in respect to vehicle A is calculated.

**Figure 12:**
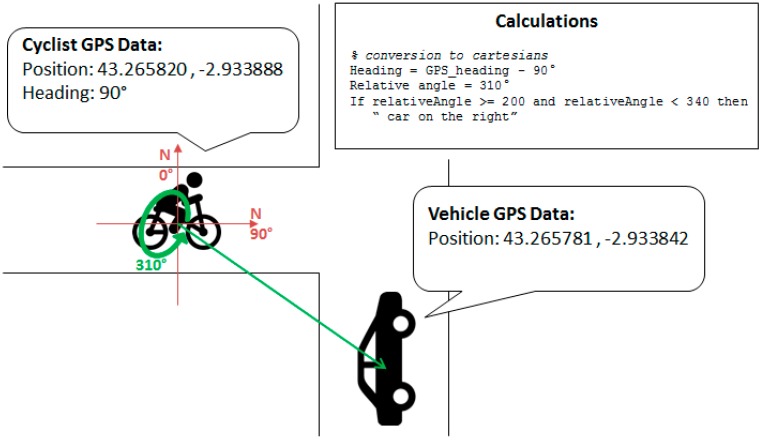
Relative position determination.

As it is described in Table 3, the heading represents the direction a vehicle is moving using the north (0°) as a reference. This information, along with the latitude, longitude, and altitude (Alt.) of the vehicle or cyclist, is provided by the GPS. First, the algorithm converts the heading and coordinates from the GPS format to the Cartesian system, where the axis that represents the north starts at 90° from the OX axis, and it is incremented anti-clockwise. This operation allows knowing the angle position of the vehicle in respect to the north. In each iteration the algorithm also checks that the calculated angle between the vehicles is between 0° and 360° to avoid negative representation of the vehicles’ angle. Then, moving the origin of the coordinates to the position of each vehicle, the angle of the other vehicle in respect to itself is calculated (Figure 12). If the vehicle is between 340° and 20°, it is considered that it is in front; between 140° and 200°, it is behind its position; between 20° and 140°, it is on its left; and between 200° and 340°, it is on its right. These calculations are carried out in each vehicle.

**Algorithm 1** Relative position algorithm description
		
**Function** *getRelative VehicularPosition(headingA, gpsPosA, gpsPosB)*
      **Data**: headingA, gpsPositionA, gpsPositionB
      **Result**: relative vehicular position
      **begin**
            *heading* ← *heading* – 90
            correctAngle (*heading*)
            *angle* ← calculateAngleBetweenTwoVehicles (*gpsPosA, gpsPosB*)
            correctAngle (*angle*)
            *relativeAngle* ← relateAngles (*heading, angle*)
            correctAngle (*relativeAngle*)

            **if** *relativeAngle is between 20 and 0 or 359 and 340* **then**
                  **return** “FRONT”
            **else if** *relativeAngle is between 140 and 200* **then**
                  **return** “BACK”
            **else if** *relativeAngle is between 21 and 140* **then**
                  **return** “LEFT”
            **else**
                  **return** “RIGHT”
            **end**
      **end**
**end**


## 5. V-Alert Validation in a Real Scenario

As it is mentioned at the beginning of this work, the purpose of V-Alert is to warn cyclists and drivers about their mutual presence. The simulations carried out in Section 3 have demonstrated how V-Alert can be deployed and operated in a large scenario where there are multiple vehicles and the communications can present more problems and restrictions. After that, in Section 4, the development of the V-Alert platform is presented and it is in this section that its reliability is tested in a real scenario. Due to the availability of only two vehicular communications gateways, this field test is smaller than the one recreated on the simulator, but it will allow us to check if it is possible to combine vehicular and mobile communications in urban scenarios and find out what the performance of V-Alert would be in this situation.

**Figure 13:**
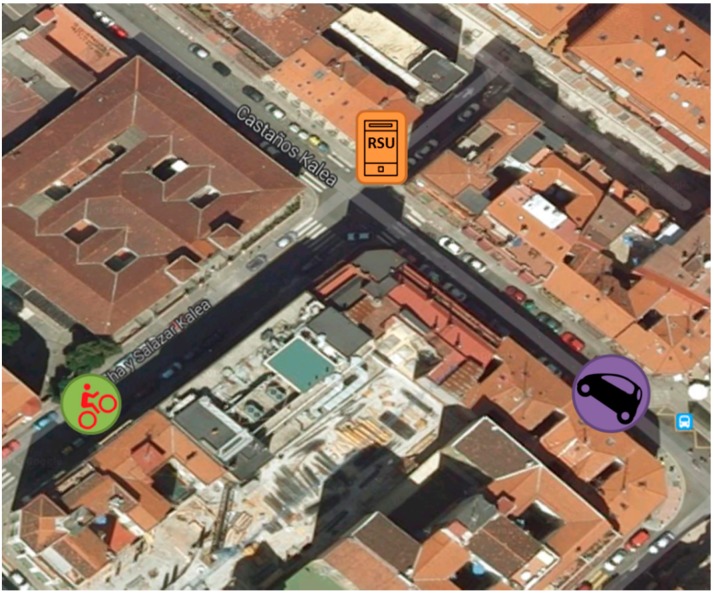
V-Alert testing scenario in the city of Bilbao.

The dependability of V-Alert relies on the communication quality between the vehicles and the cyclists, so the target of these tests is to characterize the radio communications between the vehicles and the cyclists and to obtain the probability of a cyclist/driver obtaining information about the presence of a vehicle/cyclist early enough to avoid a dangerous situation. During these trials, the worst situation has been proposed: an intersection without traffic lights and NLOS between the vehicles and the cyclist. This situation has been chosen because it is the typical one in an urban scenario where there are a lot of intersections and poor visibility. The tests were carried out in March 2015 in the city of Bilbao at the intersection of two one-way streets and with no other moving vehicles other than the ones included in the test (the test was performed during the evening). In Figure 13, the RSU is located at the intersection where the cyclist and vehicle are closing in. Both the vehicle and the RSU are equipped with a NEC Linkbird-MX IEEE 802.11p vehicular communication gateway and the RSU has a 3G interface as well. The cyclist is provided with a smartphone in which the V-Alert application has been installed. Vehicle speed in this area is restricted to 8.3 m/s and the cyclist rides up to 2 m/s, so the following calculations are focused on the vehicle whose speed is higher than the cyclist’s and then on the driver’s reaction distance because it is less than the cyclist has to avoid a collision. The position error of the GPS has not been considered in this work, but this issue is in the scope of future improvements of V-Alert.

To guarantee the success of V-Alert, the communication is characterized in two ways: first the Package Delivery Rate (PDR) in the OBU-RSU link is tested, which counts the number of sent packets from the vehicle arriving at the RSU; and second, the delay understood as the time required by a message to go from the vehicle to the cyclist. The vehicle and the cyclist send one message per second with their position. Then, safe distance (Ds) is defined as the target distance in which the vehicle and the cyclist must receive a message from the other with enough time to carry out the appropriate maneuver to avoid the collision. To calculate this distance it is necessary to take into account the driver’s reaction time and the braking distance. Using Equation (4), where *V* is the velocity expressed in Km/h, *f* represents the friction coefficient, and *i* represents the slope of the road in %, the required braking distance can be calculated [16]. Considering that the vehicle travels at 30 km/h, it requires at least 9 m to stop on a flat road. According to [17], the driver’s reaction time can be 2.3 s, during which the vehicle travels 19.09 m, so in total the driver needs 28.09 m to stop the car.

(4) $$D_{s}=\frac{V^{2}}{254\left(f+i\right)}$$

**Figure 14:**
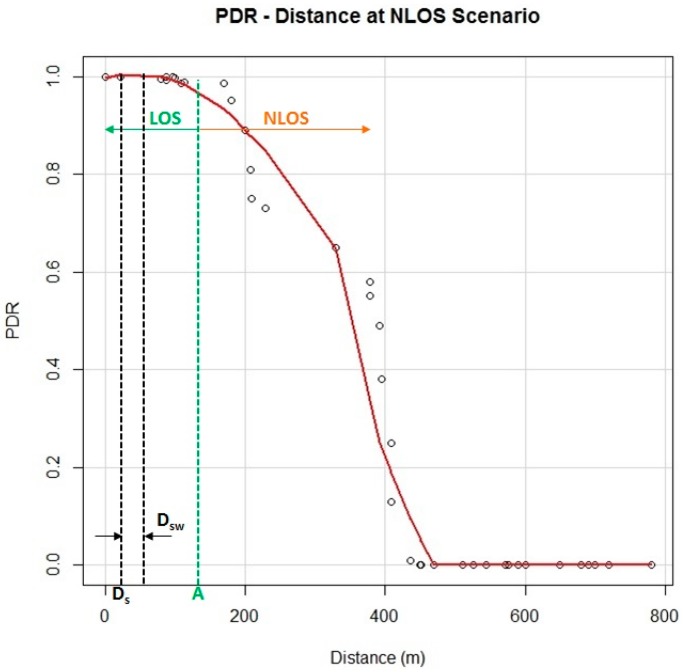
PDR measurements obtained.

In Figure 14, the PDR measurements obtained at different distances in the selected scenario are shown. The PDR has been obtained in a test in which the vehicle is approaching the RSU from a NLOS position until arriving at 175 m from the RSU (point A, Figure 14) where there is Line-of-sight (LOS) and the PDR rises to 100% when the vehicle is close to the RSU.

As the PDR is not always 1, the messages sent by the vehicle should not be exchanged only once, so they are sent many times. The easiest way would be to send the messages repeatedly until reaching the crossroad, although this is not viable since they could overload the radio link. Then, N_T_ variable is defined as the ideal number of times that a message must be sent for the application to be reliable. This variable depends on two different values: the message-sending frequency, T_f_, and the Time Safe Window (Ts_w_), which is a concept proposed to evaluate the application’s reliability. For this type of advisory application the Ts_w_ is established as 2.0 s [18].

Ts_w_ is related to D_w_, which is the distance that the vehicle travels during the time defined by Ts_w_. Considering that the vehicle speed is 8.3 m/s, in this scenario D_sw_ is equal to 16.6 m. D_sw_ distance is related to the application reliability because it needs to be defined to quantify the probability of successfully communicating between the two vehicles before the target distance Ds.

(5) $$D_{sw}=Ts_{w}*V_{speed}=\frac{N_{T}}{T_{f}}*V_{speed}$$

Therefore, the application reliability (p_app_) is defined in Equation (6) as the probability of receiving at least one packet before D_s_ in a given time Ts_w_.

(6) $$p_{app}=1-\overset{N_{T}}{\underset{k=1}{\prod }}\left(1-p_{i}\right)$$

**Figure 15:**
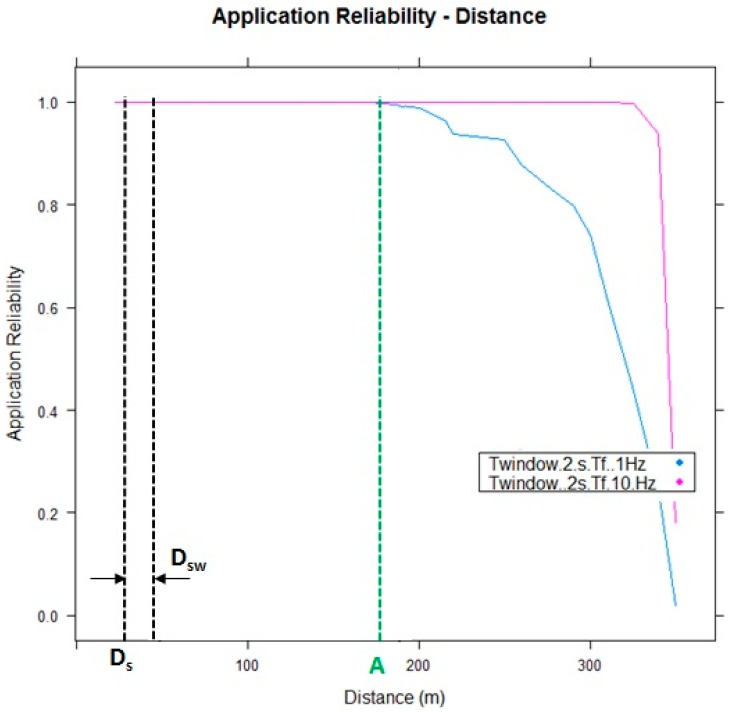
V-Alert application reliability.

Figure 15 shows the application reliability results of the performed tests, considering that the vehicle and cyclist send their positions with a frequency of 1 Hz and 10 Hz. It is shown that at point A, it means that when the vehicle enters in a LOS with the RSU, the PDR value is “1” in any case, so the V-Alert application can be considered reliable and, hence, in this scenario, the cyclist and the driver have enough time to react until an eventual accident at the intersection. Therefore, in a real and massive deployment, it will be recommended to locate the RSU at the intersections, at points with maximum visibility when the vehicles are approaching them.

During these tests, the delay in the communications has also been tested by checking the time that a message needs to go from the cyclist’s application on their smartphone to the Drivers Cloud, and the time that this message requires to arrive from the Drives Cloud to the RSU and from there to the vehicle. The results shown in Table 4 can be compared with the values obtained in the simulated scenario (Figure 2 and Figure 3). It can be seen that the results are close to similar, so it can be considered that the results obtained in the simulator can be extrapolated when V-Alert is being validated in complex scenarios. Furthermore, taking as a reference the average delays obtained in each of the links, it can be considered that the time a message requires to arrive from the cyclist to the vehicle or *vice versa* (links are symmetric) is equal to 1.0059 s. If this value is added to previous calculations, it can be seen that the application reliability has not been affected.

**Table 4:** Delay measurements in a real scenario (seconds).

Message N°	CyclistApp→DriversCloud	DriversCloud→RSU	RSU→Vehicle
1	1.278	0.417	0.038
2	0.342	0.416	0.038
3	0.436	0.415	0.039
4	0.429	0.416	0.038
5	0.430	0.417	0.040
6	0.470	0.416	0.040
7	0.453	0.417	0.040
8	0.401	0.418	0.043
9	0.399	0.417	0.045
10	0.855	0.416	0.043
**Average**	**0.549**	**0.4165**	**0.0404**

## 6. Conclusions and Future Work

In this paper, the V-Alert platform is described as a safety application to be fostered among Vulnerable Road Users (VRU) in the scope of an urban scenario as a Smart City. A Smart City is considered the ideal scenario because V-Alert has been designed to be deployed over a heterogeneous communication architecture in which vehicles that act as mobile sensors communicate their positions to elements placed on the infrastructure of the city through a dedicated radio link known as IEEE 802.11p, a wireless standard that is called to be the *de facto* interface for vehicular environments. On the other end of the communication are the VRU, who in V-Alert are considered to be cyclists, although motorcyclists or pedestrians can be also users of V-Alert. These VRU are reporting their positions to surrounding vehicles through a mobile communication, so V-Alert combines the data generated by mobile sensors (drivers and cyclists) through short- and long-distance communications in order to avoid harmful situations in which VRU are most susceptible to danger.

Both the Drivers Cloud and the users’ HMI have been designed and developed in a scalable and interoperable way in order to allow future extensions of V-Alert. HMIs provide accurate information to drivers and cyclists about their mutual distance and relative positions (front, left, right, or back) with enough time to take appropriate maneuvers during their crossing.

To know the performance of V-Alert in a scenario with many vehicles and cyclists, a virtual scenario has been simulated, obtaining a set of results that provided a baseline to design bigger real scenarios thanks to the validation of these results later in a real scenario. V-Alert has been deployed and tested in the urban area of Bilbao, obtaining results that allow the affirmation that V-Alert can work properly in the scope of a Smart City.

In future steps, a better cyclist HMI will be developed. The idea is to provide the cyclist with some wearable gadgets embedded in his/her clothes (in the gloves or the helmet) that, thanks to a personal area network communication interface, can obtain information from the V-Alert application and alert the cyclist about closing vehicles via some LEDs, sounds, or vibration systems available to the rider depending on the smartphone. Also, the positioning error of the GPS (~5 m) must be fixed, so in future steps position accuracy must be improved by including a Differential GPS system or application [19]. Moreover, we are improving the mobile application algorithm in order to discard the information of those vehicles that will not intersect with the cyclist and are not a risk for him/her. This improvement will be very useful in order to not overload the cyclist with too much information and distract him/her.
